# Pulsed radiofrequency neuromodulation of subchondral bone in knee osteoarthritis

**DOI:** 10.3389/fpain.2026.1968444

**Published:** 2026-10-21

**Authors:** José Fábio Lana, Daniel Jorge, João Protásio Netto, Alex Pontes de Macedo, Luyddy Pires, Fábio Costa, Tomás Mosaner, Pablo Sobreiro, Douglas Santos, Alexandre Teixeira, Olav Rohof

**Affiliations:** 1 Brazilian Institute of Regenerative Medicine (BIRM), Indaiatuba, São Paulo, Brazil; 2 Orthoregen International Course, Indaiatuba, São Paulo, Brazil; 3 Anna Vitória Lana Institute (IAVL), Indaiatuba, São Paulo, Brazil; 4 Max Planck University Center (UniMAX), Indaiatuba, São Paulo, Brazil; 5 Jaguariúna University Center (UNIFAJ), Jaguariúna, São Paulo, Brazil; 6 Federal University of Tocantins (UFT), Palmas, Tocantins, Brazil; 7 FC Sports Traumatology, Salvador, Bahia, Brazil; 8 Clínica de Dor, Porto, Portugal; 9 Independent Pain Practice, Amsterdam, Netherlands

**Keywords:** bone marrow lesion, intraosseous, NGF, osteoarthritis, pulsed radiofrequency, sensory nerve, subchondral bone, TrkA

## Abstract

Bone marrow lesions (BMLs) on magnetic resonance imaging (MRI) are associated with pain and structural progression in knee osteoarthritis (OA), yet no approved therapy targets the subchondral bone compartment with a demonstrated structural effect. We hypothesize that a pulsed radiofrequency (PRF) electrode positioned percutaneously within the subchondral trabecular bone of the medial tibial plateau may modulate a local sensory nerve-osteoclast-osteoblast circuit that sustains the BML, reducing both pain and lesion volume. The rationale combines four observations, most of them obtained in settings other than human OA. First, subchondral bone is innervated by peptidergic sensory fibers, which in human OA accompany vascular channels across the osteochondral junction, and in murine OA, osteoclasts induce sensory nerve growth into subchondral bone. Second, in genetic mouse models, sensory nerve ablation reduces bone volume and sensory knockout of EP4 or TrkA attenuates load-induced bone formation. Third, a PRF electrode generates electric fields of the order of hundreds of V/cm within millimeters of its tip, at least four orders of magnitude above the fields of 0.07 to about 2 mV/cm induced in bone-cell studies of pulsed electromagnetic fields (PEMF); the benchmark electroporation threshold comes from a non-neuronal cell line and requires bone-specific validation. Fourth, one uncontrolled series of ten patients with femoral head osteonecrosis shows that intraosseous PRF electrode placement is technically feasible, but it reported neither pain scores nor adverse-event data and does not establish safety. We specify a staged program: bone-specific electrothermal modeling and *ex vivo* dosimetry; a randomized, blinded, sham-controlled study in the murine destabilization of the medial meniscus model with functional neuronal readouts and targeted sensory denervation; large-animal dosimetry; and a human feasibility study followed by an external pilot randomized trial using quantitative BML volumetry. Prespecified predictions define success, failure, and inconclusive results. The nerve growth factor (NGF)-TrkA axis, the proposed mediator, carries an internal paradox: NGF appears reparative in necrotic bone but sensitizing in osteoarthritic joints, and anti-NGF treatment relieves OA pain while worsening subchondral bone and cartilage in an animal model. Determining which effect predominates is the central test of this proposal.

## 1 Introduction

Osteoarthritis affected an estimated 595 million people worldwide in 2020, knee osteoarthritis cases are projected to increase by about 75% by 2050, and among adults aged 70 years and older osteoarthritis is the seventh leading cause of years lived with disability (1). No pharmacological therapy has shown a durable structural effect on the subchondral compartment: in a meta-analysis of four randomized trials, intravenous bisphosphonates reduced a semiquantitative measure of BML size at 6 months but not at 12 or 24 months, with no effect of zoledronic acid on knee pain (Level I evidence) (2). A mechanistically distinct target is therefore of interest.

Bone marrow lesions detected on fluid-sensitive MRI sequences correspond to regions of trabecular microfracture, marrow fibrosis, and vascular change within subchondral bone; their imaging appearance, terminology, and clinical relevance, including their association with pain and structural progression, have been reviewed elsewhere (Level V evidence, narrative and consensus reviews) (3, 4). Semiquantitative MRI scoring of BMLs has been validated in the BLOKS and MOAKS systems (Level II evidence, measurement-validation studies) (5, 6), and semi-automated volumetric segmentation of BMLs shows intra-reader and inter-reader intraclass correlation coefficients of 0.96 and 0.97 (Level II evidence) (7). No intervention has been approved on the basis of reducing BML volume, and this is the gap addressed by the present hypothesis.

Radiofrequency treatment of the genicular nerves reduces chronic knee pain. A Bayesian network meta-analysis of genicular nerve radiofrequency ablation (29 studies: 13 randomized and 16 observational) ranked it above sham, intra-articular injection, and chemical neurolysis for up to 12 months (Level I evidence) (8), and in a randomized trial of 188 patients, pulsed genicular radiofrequency produced analgesia of smaller magnitude and duration than continuous ablative radiofrequency (Level II evidence) (9). The genicular target is neural and extra-articular, and no MRI data demonstrate structural bone change after genicular treatment. The present proposal differs in anatomical target, access route, intended mechanism, and measurable outcome: the electrode would be positioned within subchondral bone rather than beside a nerve.

The only published report of a PRF electrode positioned within bone is a single uncontrolled series of ten patients with steroid-induced osteonecrosis of the femoral head, reported together with a rat model (Level IV evidence) (10). A Baylis PMG-115-TD generator was used under fluoroscopic guidance to deliver PRF at 42 °C, 2 Hz, and a pulse width of 20 ms for 120 s at two to three sites within the femoral head, in weekly sessions. The report presents MRI at 13 and 23 months showing reduced bone edema in an illustrative case, and plasma NGF that was higher after treatment than before; it reports no pain or function scores and no adverse-event or complication data, and in the rat arm PRF was applied through electrodes attached to the feet rather than to bone. The series was conducted in a different disease and a different joint and had no control arm. It therefore establishes neither efficacy nor safety for the proposed indication. It supports only the narrower claim on which this manuscript relies: that intraosseous placement of a PRF electrode in weight-bearing bone has been performed and is technically feasible.

The remainder of the article sets out the anatomical and molecular basis of the hypothesis (Section 2), with each link graded in Table 1 as established, extrapolated from another setting, or proposed here; the physical rationale for an intraosseous electrode (Section 3); the NGF paradox (Section 4); procedural precedents (Section 5); and a staged program with prespecified predictions (Section 6), summarized in Table 2.

**Table 1:** Evidence map for the links in the proposed circuit. Established: demonstrated directly in the species and tissue stated. Extrapolated: demonstrated in another tissue, cell type, disease, or species and applied here by analogy. Proposed: not yet tested; this article formulates the test. Partly established: shown for part of the link only, as specified. Feasibility only: the procedure has been performed, but its effects and safety have not been assessed.

Link in the proposed circuit	Evidence and system	Status	References
CGRP-positive sensory fibers in OA subchondral bone and osteochondral channels; CGRP- and TrkA-positive subchondral bone afferents	Histology of human tibiofemoral OA; retrograde tracing in normal and OA rats	Established (human for CGRP; rat for TrkA)	(12–14, 16, 17)
Higher NGF expression and osteoclast density in BML regions	Histology of human tibial plateau	Established as an association (human)	(15)
Osteoclast-derived netrin-1 recruits sensory axons and contributes to OA pain	Cell-specific gene deletion	Established (mouse)	(18)
PGE2-EP4 signaling on subchondral osteoclasts and sensory nerves	Gene deletion and pharmacology	Established (mouse)	(22, 27)
Efferent arm: sensory EP4 and TrkA signaling, hypothalamic relay, reduced sympathetic tone, bone formation	Gene deletion, propranolol rescue, loading models	Established (mouse); not shown in OA knee or in humans	(22–24)
Local action of sensory nerves on LepR-positive stromal cells via semaphorin 3A	PEMF in aging mouse bone	Extrapolated (different stimulus and condition)	(25)
ASIC3 transduces the acidic resorption microenvironment in subchondral afferents	Myeloma bone pain (mouse); knee joint afferents in MIA (rat)	Extrapolated (not shown in subchondral afferents of human OA)	(28, 29)
Direct contact between sensory terminals and osteoclasts	Electron microscopy of rat epiphyseal trabeculae	Partly established; distance in human OA not measured	(19)
PRF fields above 330 V/cm induce calcium uptake by mild electroporation	HEK-293 cells *in vitro*	Extrapolated (not tested in neurons or bone)	(30)
Intraosseous PRF electrode placement in weight-bearing bone	Single-arm series, *n* = 10, femoral head osteonecrosis	Feasibility only; safety and efficacy not established	(10)
Change in local NGF after intraosseous PRF is reparative rather than sensitizing in OA	Reparative in osteonecrosis; sensitizing and structurally protective in OA	Proposed; central test (Phase 0)	(10, 31–33)
Intraosseous PRF reduces pain and BML volume in knee OA through sensory nerves	No data	Proposed (Stage 0A to Phase II)	This article

**Table 2:** Stages of the program, primary measurements, and prespecified decision rules.

Stage	Primary measurements	Proceeds if	Stops, redirects, or refutes if
Stage 0A: bone-specific modeling and *ex vivo* dosimetry	Finite-element field and temperature for three pulse patterns; fiber-optic thermometry and impedance in *ex vivo* tibial plateau; calcium responses in cultured DRG neurons	A pulse pattern yields a field above the neuronal threshold within the target volume without exceeding the temperature limit	No pattern reaches the neuronal threshold without thermal excess: dosimetry is revised before any *in vivo* work
Phase 0: DMM mouse model, early and established cohorts (Prediction 1)	Knee hyperalgesia (primary); non-evoked pain; ATF3 in retrogradely labeled DRG neurons and dorsal horn c-Fos; CGRP and TrkA fibers; local NGF; micro-CT; histomorphometry; OARSI and synovitis scores	With target engagement confirmed, PRF improves pain and remodeling relative to sham insertion, and the structural effect is lost after sensory denervation	CI excludes a standardized difference of 1.2 despite engagement; structural effect persists after denervation; or NGF rises with worse pain, cartilage, or synovitis
Phase 0B: large-animal dosimetry and safety, *n* = 8 (descriptive, not powered)	Temperature at fixed distances from a human-scale electrode; impedance; entry site, osteonecrosis, and marrow injury by CT, MRI, and histology at 2 and 12 weeks	No thermal or structural injury beyond the needle tract	Thermal necrosis, fracture, or marrow injury attributable to the field
Phase I: human feasibility, *n* = 12 (Prediction 2)	Serious and procedure-related adverse events at 3 months; procedural success; *in vivo* impedance; MRI of tract and entry site	No device-related serious adverse event attributable to the field; field within 2 mm of the tip above the Stage 0A threshold	Any field-related serious adverse event, or a field below the threshold: no advance without revised dosimetry
Phase II: external pilot RCT, 35 per arm (Predictions 3 and 4)	Semi-automated BML volume at 6 and 12 months; WOMAC, KOOS pain; OMERACT-OARSI responders; recruitment, retention, blinding	Difference in BML volume change favors PRF and is maintained at 12 months; pain benefit over sham	CI excludes a difference larger than the SDC (structural claim), or excludes a 15-percentage-point advantage in OMERACT-OARSI responder proportion (analgesic claim)

CI, confidence interval; SDC, smallest detectable change.

## 2 The innervated subchondral niche

### 2.1 Sensory nerves in subchondral bone

Histological and molecular studies of the tibial subchondral compartment show that it is innervated by sensory fibers, including unmyelinated C fibers and thinly myelinated A-delta fibers, together with sympathetic fibers, and that these nerves take part both in pain and in the regulation of bone homeostasis (Level III–IV evidence, review of human and animal studies) (11). Nerves enter the epiphysis mainly through the nutrient foramen and run alongside the arteries; the fibers identified in subchondral bone are TrkA-, calcitonin gene-related peptide (CGRP)- and substance P-positive, and in OA nerves and vessels grow together into osteochondral junctions after tidemark penetration (11). In human tibiofemoral OA, substance P- and CGRP-positive sensory fibers and sympathetic fibers accompany vascular channels that breach the osteochondral junction, in mild as well as severe disease (Level IV evidence, human tissue) (12). Osteochondral channels containing CGRP-positive nerves are more frequent in symptomatic than in asymptomatic chondropathy, NGF expression in osteochondral channels and subchondral osteoclast density are higher in symptomatic tibial plateaus (13, 14), and regions of the tibial plateau with a BML show higher NGF expression and osteoclast density than regions without one (Level IV evidence, human tissue) (15). In rats, subchondral bone afferents identified by retrograde tracing are predominantly CGRP- and TrkA-positive, and their nociceptive phenotype intensifies as experimental OA progresses (Level III evidence) (16, 17).

In murine OA, osteoclasts at sites of aberrant subchondral remodeling secrete netrin-1, which induces sensory axonal growth into subchondral bone (Level III evidence, murine model) (18). Sensory innervation therefore expands where resorption is active. Direct contact between CGRP-positive fibers and osteoclasts has been shown by electron microscopy in rat epiphyseal trabeculae (Level III evidence) (19). The cellular-scale proximity of sensory terminals to individual osteoclasts, perivascular stromal cells, and vessels shown schematically in Figure 1 is inferred from these data and has not been quantified in osteoarthritic human bone; quantifying it is one of the Phase 0 histological readouts. This proposed niche is the anatomical substrate of the hypothesis: an intervention delivered at this site could act on both the nociceptive and the remodeling elements.

**Figure 1:**
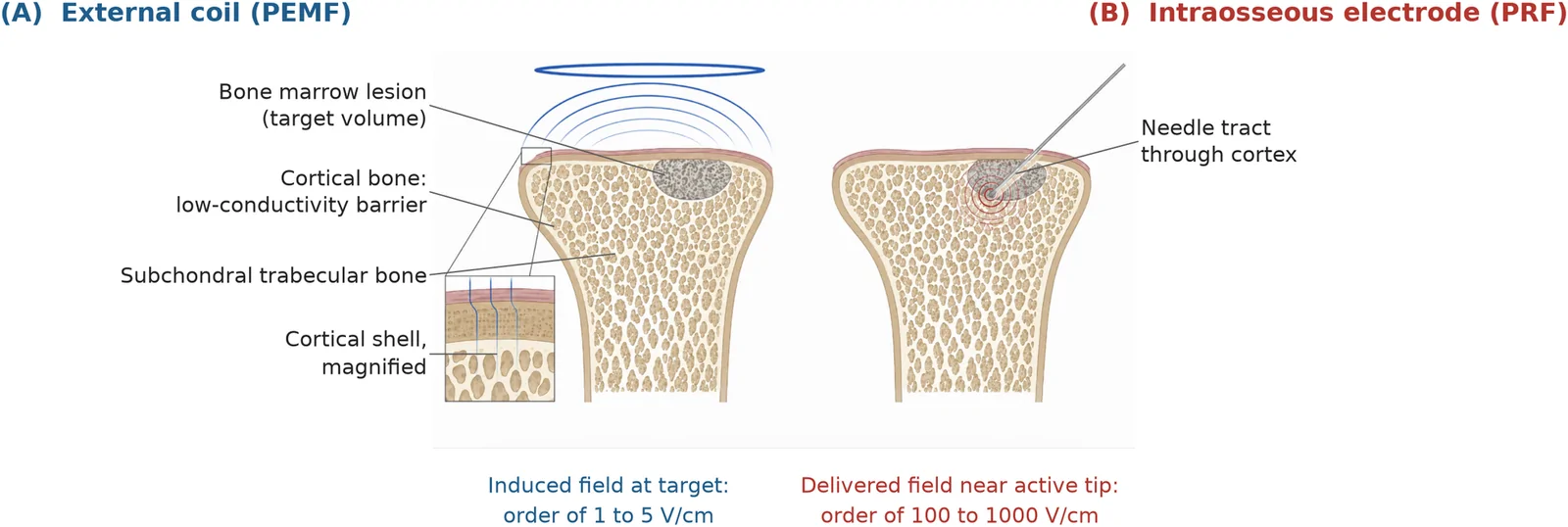
The subchondral neurovascular niche (proposed anatomical substrate). Schematic cross-section of the human medial tibial plateau from articular cartilage (top) to subchondral trabecular bone (bottom), showing articular cartilage, tidemark, calcified cartilage, cement line, subchondral bone plate, and trabecular bone with marrow spaces. Unmyelinated sensory C fibers (orange) ascend through the marrow spaces and terminate close to three cellular targets: a multinucleated osteoclast in a Howship resorption lacuna, a leptin receptor-positive (LepR+) perivascular stromal cell, and a type H vessel. An osteoblast row with an osteoid seam and osteocytes with their canalicular network are also shown. Sensory innervation of tibial subchondral bone, its association with vascular channels at the osteochondral junction in human OA, and its osteoclast-induced growth in murine OA are described in (11, 12, 18); direct contact between CGRP-positive fibers and osteoclasts has been shown in rat bone (19), whereas the cellular-scale proximity to LepR+ cells and type H vessels shown here is inferred rather than quantified, and the figure is a schematic that does not represent a specific histological specimen. The drawing is not to scale. LepR, leptin receptor.

For broader context, direct bone-neural interfaces are found elsewhere in the skeleton. Calvarial bone channels have been proposed as a putative craniomaxillary-brain interface in central nervous system immunity (20), and craniomaxillofacial bone marrow stromal cells retain a neural crest-derived neurogenic niche relevant to cognitive aging (21). These findings concern different skeletal sites, cell populations, and diseases, and are cited only to show that bone-neural conduits are a recurring skeletal feature, not as evidence for the circuit proposed here.

### 2.2 The sensory nerve as a regulator of bone formation

Proximity alone does not establish function. In genetic mouse models, ablation of sensory nerves erodes skeletal integrity, and knockout of the prostaglandin E2 (PGE2) receptor EP4 in sensory neurons, or of cyclooxygenase-2 in osteoblastic cells, reduces bone volume in adult mice (Level III evidence) (22). In a related line of work, load-induced bone formation involved hypothalamic processing of skeletal PGE2 signaling, and knockout of EP4 or of TrkA in sensory nerves (23), or pharmacogenetic inhibition of TrkA signaling (24), attenuated the osteogenic response to mechanical loading (Level III evidence).

These data support the interpretation that sensory fibers in bone are bidirectional: they report the local mechanical and chemical state centrally, and the resulting signal regulates bone formation. In these models, the efferent limb that has been demonstrated is central rather than local: skeletal PGE2 sensed through EP4 on sensory nerves is processed in the hypothalamus, which lowers sympathetic tone, and beta-adrenergic blockade with propranolol rescued the bone loss that followed sensory denervation (22, 23). A direct local trophic action of sensory terminals on osteoblastic progenitors and stromal cells has been shown for PEMF (below), not for this homeostatic relay. This bidirectionality has been shown in murine models of bone homeostasis and mechanical loading. It has not been shown in osteoarthritic bone carrying a BML, and this manuscript treats that extension as the central unproven step of the hypothesis rather than as an established fact.

PEMF provides a further precedent. In aging male mice, PEMF exposure increased bone formation and innervation; the effect required intact sensory nerves and was abolished by sensory nerve depletion or by knockout of semaphorin 3A (Sema3A), which sensory nerves secrete and which acts through neuropilin-1 on leptin receptor-positive stromal cells (Level III evidence, murine model) (25). This shows that a pulsed electromagnetic stimulus can promote osteogenesis by using the sensory nerve as an obligatory relay. It is not evidence that PRF, a stimulus of different physics and field magnitude (Section 3), engages the same relay, nor that it does so in osteoarthritic bone. Three inferential steps therefore remain to be tested rather than assumed: that a sensory-nerve relay of this kind operates in osteoarthritic subchondral bone; that PRF engages it despite its different field regime (Section 3); and that engaging it produces net osteogenesis rather than net sensitization in a joint where NGF signaling is already elevated (Section 4). The Phase 0 study in Section 6 tests all three steps directly, with functional neuronal readouts and a targeted sensory denervation arm, because changes in NGF or receptor immunolabeling alone cannot establish causality.

### 2.3 Local acidification and the osteoclast-nerve circuit

Osteoclasts secrete protons into the resorption lacuna to dissolve bone mineral, and the resulting acidic microenvironment has been linked to bone pain in tumor-bearing bone (Level III evidence, rat model) (26). Local remodeling is coupled to the sensory nerve through two further signals. First, osteoclasts secrete netrin-1, which induces sensory axonal growth into subchondral bone; osteoclast-specific knockout of netrin-1, or knockdown of its receptor DCC, reduces pain behavior in a murine OA model (Level III evidence) (18). Second, PGE2 released during remodeling activates EP4, which is expressed on osteoclasts, where it promotes osteoclastogenesis and OA progression, and on sensory nerve terminals, where it links skeletal remodeling to a hypothalamic relay that regulates bone formation (Level III evidence) (22, 23, 27). Acid-sensing ion channel 3 (ASIC3) mediates proton-driven bone pain in other contexts: in a murine model of multiple myeloma bone pain, CGRP-positive sensory nerves in bone upregulated ASIC3, and an ASIC3 antagonist reduced pain behavior (Level III evidence) (28); in the rat monosodium iodoacetate model of OA, ASIC3 increased in knee joint afferents, and intra-articular ASIC3 blockade reduced weight-bearing asymmetry (Level III evidence) (29). ASIC3 expression in subchondral bone afferents of osteoarthritic joints has not been demonstrated, and its inclusion here is a plausible extension rather than an established finding.

Taken together, these signals place the ascending nociceptive traffic that may explain why joints with BMLs are painful, and the descending signals that regulate osteoclastic and osteoblastic activity, at a shared anatomical and molecular interface. The result is a proposed circuit rather than a unidirectional pathway: osteoclastic activity activates the sensory terminal, the terminal signals centrally, and efferent signals, demonstrated in mice as a hypothalamic relay acting through sympathetic outflow, modulate resorption and formation. This architecture is shown in Figure 2. If an intervention delivered at the terminal reduced its responsiveness to protons and PGE2, it could in principle reduce pain and shift the balance between resorption and formation in a direction favorable to BML regression. This is the mechanistic claim under test, not an established mechanism. Table 1 classifies each link of the proposed circuit as established, extrapolated, or proposed, with the species or system in which it was shown.

**Figure 2:**
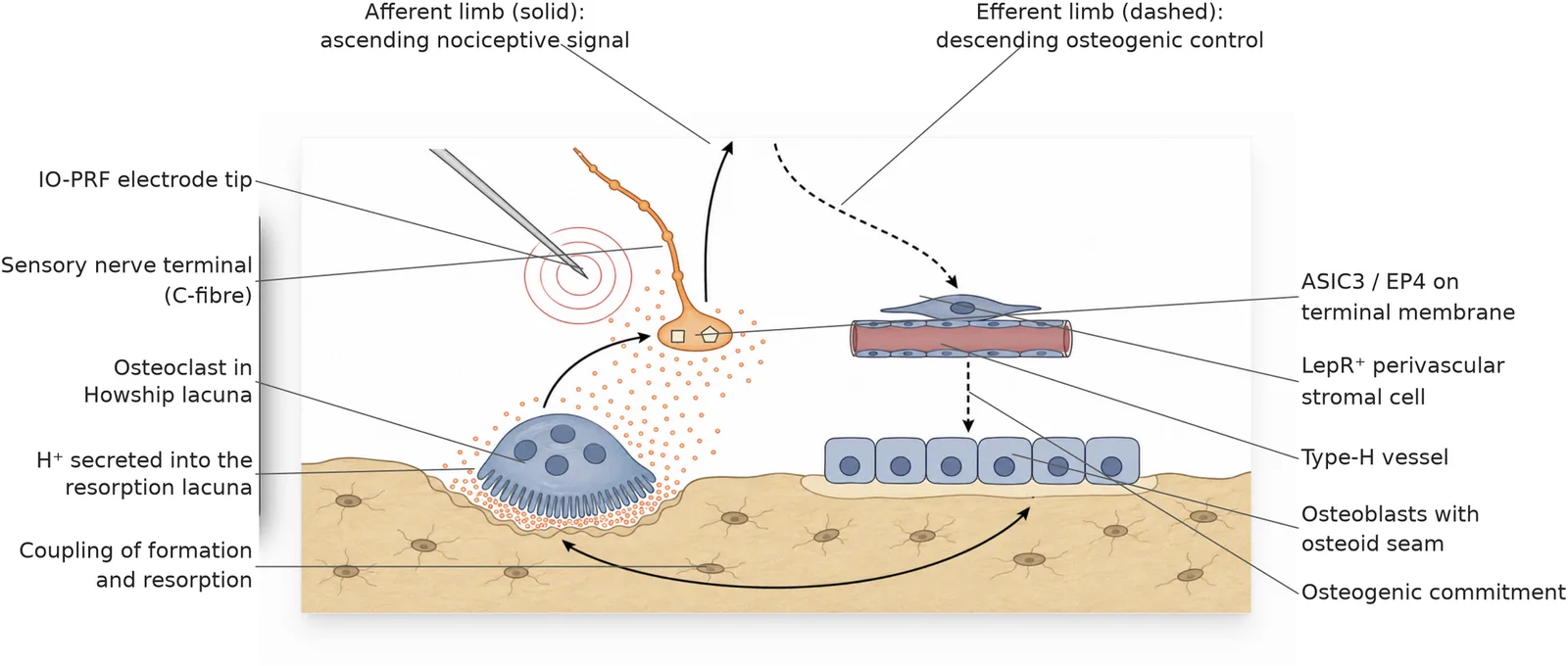
The proposed osteoclast-nerve-osteoblast circuit and the point of intervention. Osteoclasts in the resorption lacuna secrete protons (H+, red dots) and netrin-1, which induces sensory axonal growth (18); prostaglandin E2 released during remodeling activates EP4 receptors on osteoclasts and on sensory terminals (22, 23, 27). The terminal membrane is labeled with EP4 and, as a candidate proton sensor, ASIC3, which has been demonstrated in myeloma bone pain and in knee joint afferents in a rat OA model (28, 29) but not in subchondral bone afferents in OA. The afferent limb (solid arrow) carries the nociceptive signal centrally; the efferent limb (dashed arrow) represents proposed descending control acting on the LepR+ perivascular stromal cell adjacent to the type H vessel, promoting osteogenic commitment and osteoblast activity; in mice, the efferent pathway that has been demonstrated is a hypothalamic relay acting through reduced sympathetic outflow (22, 23), and its local action on LepR+ cells is proposed. The curved double arrow at the base represents coupling of resorption and formation. The intraosseous PRF electrode tip (concentric red rings) is placed at the sensory terminal, the proposed point of intervention. Solid lines denote links established in published animal data; dashed lines denote links proposed in this hypothesis. ASIC3, acid-sensing ion channel 3; EP4, prostaglandin E2 receptor 4; IO-PRF, intraosseous pulsed radiofrequency; LepR, leptin receptor.

## 3 Why an intraosseous PRF electrode is not equivalent to external PEMF

PEMF and PRF both use pulsed waveforms that induce electric fields in tissue, but they differ in physics and in the magnitude of the field delivered. Bone-cell studies of PEMF report induced electric fields of the order of 2 mV/cm, and in one osteoblast culture study the most effective intensity was about 2 mV/cm, with higher intensities not producing greater growth (Level IV evidence, *in vitro*) (34, 35). For the 75 Hz, 1.5 mT signal used clinically in orthopedics, the induced field measured with a standard coil probe *in vitro* was 0.07 mV/cm (Level IV evidence) (36). PEMF effects on osteogenesis and chondrogenesis have been attributed to receptor-mediated signaling, notably adenosine receptor signaling (Level III evidence, *in vitro* and animal studies) (37). The attenuation of PEMF power by cortical bone has been measured directly: in blood and cortical bone samples, power attenuation at the frequencies tested did not exceed about 1 dB (Level IV evidence, experimental and theoretical dosimetry) (38). External PEMF is not biologically inert in subchondral bone: in an uncontrolled series of 28 patients with early spontaneous osteonecrosis of the knee, 90 days of PEMF was followed by reduced BML area on MRI (Level IV evidence) (39). The intraosseous argument therefore does not rest on cortical bone blocking an external field. It rests on field magnitude and focality: an external coil induces a weak, distributed field, whereas an electrode delivers a strong field confined to a small volume around its tip.

A radiofrequency electrode in tissue generates its highest electric fields close to the active tip, and finite-element models, which agreed substantially with *ex vivo* tissue measurements, show that pulsed radiofrequency produces very high local fields and transient temperature spikes during each burst (Level IV evidence, computational and *ex vivo* study) (40). Fields of up to 250,000 V/m (2,500 V/cm) have been reported in a thin layer around an invasive needle tip, compared with 50–250 V/m for transcutaneous, whole-compartment PRF applications (Level IV evidence) (41). *In vitro*, PRF bursts produced calcium influx at fields above 480 V/cm after a single burst and at 330 V/cm after a train of 120 bursts, with a temperature increase below 5 °C (Level IV evidence) (30). These thresholds were obtained in HEK-293 cells, a non-neuronal cell line, and have not been validated in sensory neurons, marrow, or trabecular bone. They are used here only as order-of-magnitude benchmarks for the field an electrode can produce near its tip, not as validated biological thresholds. Mild electroporation, a transient non-lethal permeabilization of the plasma membrane, is therefore treated as a working hypothesis for the mechanism at the terminal.

On this basis, a PRF electrode produces fields of the order of hundreds of V/cm within millimeters of its tip, at least four orders of magnitude above the fields induced in PEMF bone-cell studies. Whether such fields reach and modulate sensory terminals within trabecular bone depends on the dielectric properties of marrow and mineralized tissue, which differ from those of the soft tissues in which radiofrequency fields have been modeled. Bone-specific electrothermal modeling, *ex vivo* dosimetry in trabecular bone, and a dose-response study in a neuronal preparation are therefore specified as prerequisites to any human application (Section 6, Stage 0A), and Prediction 2 is framed around the possibility that the benchmark field is not reached in living bone.

Two published parameter sets operate under the 42 °C limit. In the osteonecrosis series, PRF was delivered at 2 Hz with a 20-ms pulse width, a duty cycle of 4.0% (10). A prospective series combining transforaminal PRF with platelet-rich plasma for radicular pain with grade I lumbosacral spondylolisthesis used 500 kHz, a 5-ms pulse width at 5 Hz, 240 s, and 40 V, with a 42 °C tip-temperature limit, a duty cycle of 2.5%; no severe complications were reported at 6 months in 96 patients (Level IV evidence) (42). Both configurations use generator temperature monitoring at the tip; neither study measured temperature within bone at a distance from the tip, and transient temperature spikes during each burst exceed the average tip reading (Level IV evidence) (40).

Pulse pattern can reduce these spikes. In a computer model checked against an agar phantom, a protocol of 5-ms pulses at 5 Hz and higher voltage increased the electric field in tissue by about 20% without increasing temperature, compared with the standard 20-ms, 2-Hz protocol, and the temperature controller was the main factor preventing thermal damage (Level IV evidence, computational and phantom study) (43). The Sluijter-Teixeira-Poisson (STP) mode goes further: it delivers short pulses at stochastic, Poisson-distributed intervals with an average duty cycle slightly below 1.5% (44). An international consensus considers a duty cycle below 2.5% and a 5-mm active tip optimal to prevent heat spikes, and recommends STP for intra-articular application at 3 or 5 Hz, 5 ms, and 40 V, for 8 min in small joints and 12 min in large joints (Level V evidence, expert consensus) (44). The consensus states explicitly that this recommendation rests on expert experience and theoretical considerations rather than on robust comparative evidence. Two retrospective series of intra-articular STP in knee OA, of 39 and 129 patients, reported pain reduction, with no complications in one and no major adverse events in the other (Level IV evidence) (45, 46). No study has measured tissue temperature during STP, compared peak temperatures between STP and fixed-pattern PRF, or applied STP within bone. STP is therefore a candidate for minimizing thermal load in the intraosseous setting, not a demonstrated solution.

Thermal behavior in bone has to be measured rather than assumed. Stage 0A (Section 6) therefore compares the peak temperature at fixed distances from the tip for three patterns at equivalent field strength: 2 Hz with 20 ms, the only pattern used so far within bone (10); 5 Hz with 5 ms; and STP. The pattern with the lowest peak temperature at an adequate field is carried into Phase 0 and Phase I.

A separate hypothesis proposes that PRF acts in part through its magnetic-field component, favoring radical-pair recombination and dampening an oxidative inflammatory cycle, termed RedoxPRF (Level III–IV evidence) (41). This mechanism is not exclusive of electroporation, because each PRF burst generates both electric and magnetic fields, but the two have not been experimentally dissociated, and RedoxPRF was described at transcutaneous rather than peri-electrode field intensities. It is recorded here as a second candidate mechanism that the Phase 0 design does not isolate.

Two implications follow. First, effects attributed to PEMF cannot be transferred to PRF without evidence that the same pathway operates at PRF field intensities. Second, an electrode positioned within trabecular bone delivers its field directly into the compartment that contains the lesion, whereas an external coil delivers a weaker field distributed across skin, soft tissue, cortex, and marrow (Figures 3A,B). This geometric argument justifies the intraosseous route; it is not a claim that the resulting field is known to be therapeutic.

**Figure 3:**
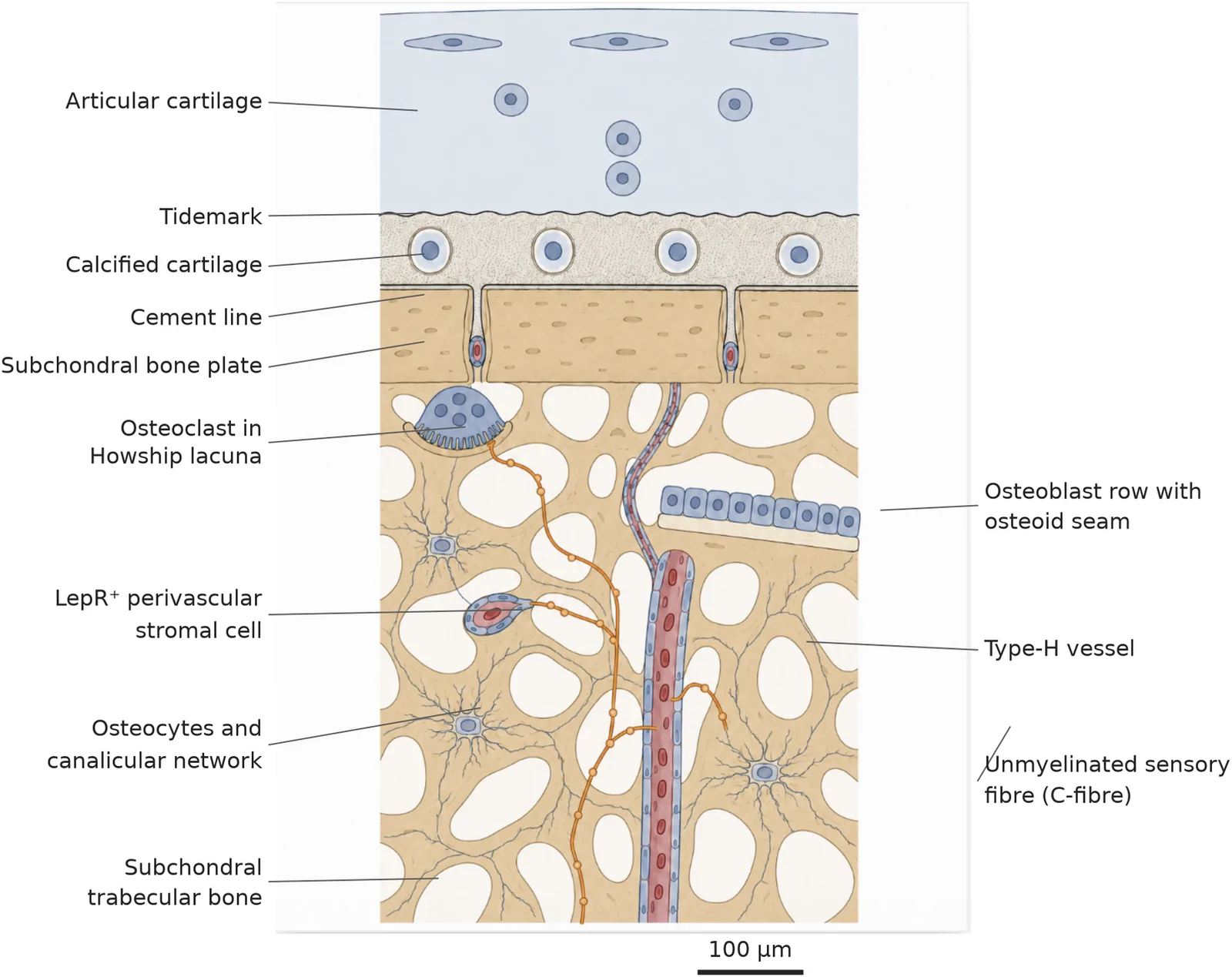
Field delivery by an external PEMF coil compared with an intraosseous PRF electrode. Both panels show the same schematic proximal tibia, with the target bone marrow lesion drawn with the same neutral fill, so that color encodes only the energy modality. **(A)** An external PEMF coil (blue) induces a weak field distributed across skin, soft tissue, cortical bone, and marrow; the inset magnifies the cortical shell. Bone-cell studies of PEMF report induced fields of the order of mV/cm or less, from 0.07 mV/cm for the clinical 75 Hz, 1.5 mT signal to about 2 mV/cm (34–36), and measured attenuation of PEMF power by cortical bone does not exceed about 1 dB (38); the difference between the panels therefore lies in field magnitude and focality, not in a cortical barrier. **(B)** A PRF electrode passes through a small cortical perforation and delivers a focal field (red contours) within the trabecular compartment, with fields of the order of 100–1,000 V/cm within millimeters of the tip (30, 40, 41). Field magnitudes are order-of-magnitude estimates from soft-tissue and *in vitro* data; the field distribution within human trabecular bone has not been measured, and its measurement is the purpose of Stage 0A, Phase 0B, and Phase I. PEMF, pulsed electromagnetic field; PRF, pulsed radiofrequency.

## 4 The NGF paradox

Nerve growth factor is the principal survival and sensitization signal for TrkA-expressing sensory neurons. In patients with hip or knee OA, subcutaneous tanezumab, an anti-NGF antibody, improved pain and function over 24 weeks in a phase III randomized trial (Level I evidence) (32). The standard interpretation is that NGF lowers the firing threshold of sensory terminals and promotes the sprouting of additional fibers. Anti-NGF treatment, however, also worsened subchondral bone and cartilage measures while improving symptoms in rabbits with experimental OA (Level III evidence) (33). In the clinical anti-NGF programs, rapidly progressive OA occurred, with risk concentrated at higher doses, with concomitant use of nonsteroidal anti-inflammatory drugs, and in joints with pre-existing subchondral insufficiency fractures (Level II evidence, adjudication of trial events) (47). In OA, the analgesic and the structural effects of NGF signaling can therefore move in opposite directions. Section 2 treats NGF-TrkA signaling as the nociceptive arm of the proposed circuit, while recognizing that its structural role has not been separated from its nociceptive role in any clinical data.

In the osteonecrosis series, plasma NGF was higher after intraosseous PRF than before, NGF neutralization counteracted the effect of PRF in the rat arm, and the authors proposed NGF elevation as the mechanism of repair (Level IV evidence, *n* = 10) (10). In resected human osteonecrotic tissue, NGF is concentrated in the reparative zone around the necrotic core, localized to osteoblast-like and stromal cells, where its expression correlates with appositional bone formation and with osteogenic gene expression (Level IV evidence; two publications from the same group, representing convergent confirmation by different methods rather than independent replication) (31, 48). A rabbit model of steroid-induced osteonecrosis from an independent group showed changes in sensory and sympathetic fibers and in NGF immunoreactivity that were temporally associated with the development of necrosis (Level III evidence) (49).

This produces a direct contradiction: in osteonecrosis NGF elevation appears reparative, whereas in OA it is the target of analgesic blockade, and its blockade can worsen bone and cartilage structure. The answer for subchondral bone carrying a BML is unknown. The osteonecrosis series measured plasma rather than local NGF, and plasma levels reflect systemic sources. The Phase 0 study (Section 6) therefore measures local NGF distribution and sensory nerve sprouting together with cartilage integrity, synovitis, and pain behavior, because a change in NGF in either direction is compatible with a favorable or an unfavorable outcome depending on the concurrent effect on cartilage and pain. If local NGF increases while pain behavior worsens or cartilage and synovial measures deteriorate, the therapeutic hypothesis fails for mechanically driven OA. If local NGF increases while pain behavior improves and cartilage and synovial measures do not deteriorate, the reparative context predominates. If NGF decreases, the mechanism differs from that proposed for osteonecrosis and must be recharacterized.

Treatment timing. Part of the paradox may be a question of timing. Bone-innervating sensory neurons appear to be temporally plastic: after skeletal injury they shift from an early nociceptive state to a later pro-regenerative secretory program, a transition that tracks the stage of repair rather than a fixed interval and varies with site, age, and injury context; broad neural inhibition may relieve pain while compromising osteogenesis (Level V evidence, review of predominantly fracture-healing studies) (50). If a comparable window exists in the chronic lesion represented by a BML, the aim would be to time or titrate PRF so that suppression of nociceptive signaling does not abolish regenerative neural output. Whether this fracture-healing framework applies to a chronic degenerative lesion is itself untested. Phase 0 therefore compares treatment at an early and at an established stage of the DMM model and measures outcomes at several timepoints (Section 6).

## 5 Precedents for energy delivery within bone

Positioning an electrode within subchondral bone is unconventional but has procedural precedents.

Basivertebral nerve ablation uses a bipolar radiofrequency probe inserted into the vertebral body under fluoroscopic guidance. A randomized, double-blind, sham-controlled trial of 225 patients and an open-label randomized multicenter trial of 140 patients against standard care reported improvement in function and pain in chronic low back pain (Level I and Level II evidence, respectively) (51, 52). The target is thermal ablation, not neuromodulation, and the energy delivered far exceeds PRF; these trials show that intraosseous radiofrequency delivery in the axial skeleton can be performed under controlled trial conditions.

Electrical stimulation for femoral head osteonecrosis uses electrodes or coils to promote bone formation. A systematic review of ten studies found that pulsed electromagnetic fields gave the best responses in early stages and that outcomes with direct-current and capacitive coupling were more ambiguous (Level III–IV evidence, systematic review of eight prospective and two retrospective studies) (53), and a retrospective series of 60 patients (66 hips) treated with invasive electrical osteostimulation, in which a stimulating screw electrode remained in the femoral head for 3 months, reported no complication attributable to the stimulation device or procedure over a mean follow-up of 58 months (range 19–110) (Level IV evidence) (54). The field parameters differ from PRF, but an electrode can remain within the femoral head for 3 months without device-related complications in the reported series.

Intraosseous injections for knee BMLs are the closest procedural comparators. In a randomized, double-blind trial, intraosseous plasma rich in growth factors (PRGF; 42 patients analyzed) produced greater improvement in pain and function at 12 months than intraosseous saline (41 analyzed), both combined with intra-articular PRGF, with total WOMAC in the PRGF arm falling from 60.3 to 33.5 and no serious adverse reactions reported (Level I evidence) (55). The saline arm also improved, but because it received intra-articular PRGF, the contribution of intraosseous access alone cannot be separated, and the trial did not include follow-up MRI. A prospective multicenter pilot cohort of combined intra-articular and subchondral bone marrow aspirate concentrate (*n* = 30; MRI analyzed in 28 knees) showed a reduction in the bone marrow edema component of the WORMS score at 12 months, with other WORMS parameters unchanged and no major complications (Level IV evidence, single-arm pilot) (56). Subchondroplasty, the fluoroscopically guided injection of calcium phosphate into BMLs, was associated with clinically important improvement in pain and function in a meta-analysis of 12 studies and 829 patients, with a median complication rate of 6.7%, most often calcium phosphate leakage; the authors note that the evidence is limited by short follow-up and the lack of control groups (Level IV evidence, meta-analysis of uncontrolled studies) (57); reported complications also include postoperative pain, infection, wound drainage from extravasation, and avascular necrosis in the talus (Level IV evidence, review) (58).

These precedents show that percutaneous intraosseous access to the tibial plateau under image guidance is feasible and that energy can be delivered within bone at levels above PRF. They also define the complications to be monitored. None of them, individually or together, constitutes a safety demonstration for an intraosseous PRF electrode in OA; they establish feasibility and motivate, rather than replace, the staged safety evaluation in Section 6.

## 6 A staged translational program with testable predictions

The program has five stages, summarized with their predictions and decision rules in Table 2, followed by a conditional confirmatory trial (Phase III). Each generates a prespecified prediction that, if not met, terminates or redirects the program. Negative results at pilot scale are interpreted with confidence intervals rather than with a binary significance threshold: a result whose 95% confidence interval includes both no effect and a clinically meaningful effect is classified as inconclusive, not as refutation, and leads to a dose-response or larger study rather than abandonment. A negative result counts against the hypothesis only when target engagement has been demonstrated (see below), so that an absence of effect cannot be explained by failure to deliver an adequate field to the target.

### 6.1 Stage 0A: bone-specific modeling and *ex vivo* dosimetry

Before any *in vivo* work, a finite-element electrothermal model of the electrode in cortical bone, trabecular bone, and marrow, using measured dielectric properties at the generator frequency, will estimate the volume of tissue exposed to fields above the provisional 330 and 480 V/cm benchmarks (30) and the peak tissue temperature, following the finite-element approaches used for soft tissue (40, 43), for three pulse patterns: 2 Hz with 20 ms, 5 Hz with 5 ms, and STP (Section 3). The model will be calibrated in fresh *ex vivo* human or ovine tibial plateau specimens with fiber-optic thermometry at known distances from the tip and impedance measurement. A dose-response study in cultured dorsal root ganglion neurons, measuring calcium responses across field strengths and burst numbers, will replace the HEK-293 benchmark with a neuronal one. Output of Stage 0A: a dose specification (pulse pattern, field at a given distance, maximum temperature) to be carried into Phase 0 and Phase I; the selected pattern is the one with the lowest peak temperature at a field above the neuronal threshold.

### 6.2 Phase 0: proof of mechanism in the destabilization of the medial meniscus model

Model and timeline. The murine destabilization of the medial meniscus (DMM) model is used because it combines surgical joint destabilization with quantifiable, stage-dependent pain behavior and well-described histopathology; mechanical allodynia develops early, movement-provoked pain and DRG neuroinflammatory changes are established by about 8 weeks and persist to 16 weeks, and gene-expression signatures distinguish post-surgical, early, and persistent OA pain phases (Level III evidence) (59–61). Bone marrow lesion-like changes in rodent joints are less well characterized than in humans, which is a limitation of all small-animal BML models (Level IV evidence, review) (62); micro-CT and histology of the subchondral plate and trabeculae are therefore used as the structural readouts in mice. Because sustained pain behavior and pathology are established only by about 8–12 weeks, treatment is delivered in two cohorts: an early cohort treated at 4 weeks after DMM and an established-disease cohort treated at 12 weeks. Outcomes are measured 2, 4, and 8 weeks after treatment (that is, up to 12 weeks after surgery in the early cohort and 20 weeks in the established cohort; because DMM pain has been characterized only to 16 weeks, the DMM-without-intervention group defines the natural course at each timepoint), with separate animals at each timepoint for terminal measures. The early cohort tests whether treatment modifies the development of pathology; the established cohort tests whether it reverses existing changes, including trabecular architecture.

Electrode and parameters. A 27-gauge insulated electrode with a 0.5-mm active tip, sized to the murine proximal tibia, will be placed percutaneously into the medial tibial plateau under fluoroscopic guidance, with tip position confirmed by micro-CT at sacrifice. Parameters follow the Stage 0A dose specification (pulse pattern selected among 2 Hz with 20 ms, 5 Hz with 5 ms, and STP), with tip temperature limited to below 42 °C and impedance recorded throughout. A dose-response arm (the Stage 0A dose and half its field strength) is included so that a null result at one dose cannot be mistaken for absence of mechanism.

Groups, controls, randomization, and blinding. Seven groups per cohort: DMM plus intraosseous PRF at the Stage 0A dose; DMM plus intraosseous PRF at half the Stage 0A field strength (dose-response arm); DMM plus sham electrode insertion (identical needle, positioning, duration, and anesthesia, with no energy) to control for marrow decompression and microfracture caused by the insertion itself; DMM plus PRF after targeted sensory denervation (local resiniferatoxin applied to the medial tibial plateau before treatment, with denervation verified by loss of CGRP-positive fibers in subchondral bone); DMM plus targeted sensory denervation without PRF, because sensory denervation itself has been reported both to attenuate and to accelerate experimental OA (Level III evidence) (63, 64); sham surgery plus PRF, to test for adverse effects on normal bone; and DMM without intervention. Allocation is by computer-generated block randomization stratified by sex; surgeons are not blinded to the procedure, but all outcome assessors (behavior, imaging, histology, electrophysiology) are blinded to group. Both sexes are included. The studies will be preregistered and reported according to the ARRIVE 2.0 guidelines (65).

Sample size. The primary outcome is knee hyperalgesia measured with a pressure application device. A sample of 12 animals per group gives 80% power at a two-sided alpha of 0.05 to detect a standardized difference of 1.2 between PRF and sham insertion; allowing for about 15% attrition, 15 animals per group will be allocated (12/0.85 = 14.1, rounded up). Sex is balanced within groups; the study is not powered for sex interactions, which will be reported descriptively. The assumed effect size is provisional and will be updated from a feasibility run before the main study. This calculation applies to the primary behavioral comparison, measured longitudinally in all animals; terminal tissue measures at 2 and 4 weeks after treatment use satellite subgroups of 6 animals per group.

Target engagement and causality. Target engagement is verified in every animal by recorded impedance and temperature, by micro-CT confirmation of tip position, and in a subset by activating transcription factor 3 (ATF3) immunolabeling of retrogradely labeled ipsilateral L3 to L5 dorsal root ganglion (DRG) neurons and c-Fos immunolabeling of dorsal horn laminae I and II after treatment; in rats, PRF applied adjacent to the DRG increases ATF3 in small and medium DRG neurons, and PRF to the DRG increases c-Fos in dorsal horn laminae I and II (Level III evidence) (66, 67). Functional neuronal effect is measured by *in vivo* calcium imaging or *ex vivo* recording of knee-innervating DRG neurons in response to intra-articular or intraosseous stimuli, together with retrograde labeling to identify bone-innervating neurons (16). The denervation arm provides the targeted perturbation. Causality is tested as the interaction between PRF and denervation, comparing PRF plus denervation with denervation alone, and PRF with sham insertion. Because resiniferatoxin is itself analgesic, this interaction test applies to the structural and nerve outcomes, and the dependence of any analgesic effect on sensory nerves is inferred from the target-engagement and DRG readouts. If the structural effects of PRF persist after local sensory denervation, they do not depend on the sensory nerve, and the proposed mechanism is not supported even if the effects are beneficial.

Outcomes. Pain: knee hyperalgesia (pressure application measurement) (68), mechanical allodynia (von Frey), and non-evoked measures [weight-bearing asymmetry (69), gait analysis, and burrowing (70)]. Nerve: density and sprouting of CGRP-positive and TrkA-positive fibers in subchondral bone, local NGF protein and distribution, ASIC3 and EP4 immunolabeling; and the distance from CGRP-positive terminals to the nearest osteoclast, LepR-positive stromal cell, and type H vessel. Bone: micro-CT trabecular bone volume fraction, trabecular thickness, and subchondral plate thickness; histomorphometric osteoclast and osteoblast surface fractions. Joint safety: OARSI cartilage score and synovitis score. Thermal safety: histological necrosis around the electrode tract.

Adverse-event criteria. Prespecified humane and stopping criteria include tibial fracture, infection, osteonecrosis or thermal necrosis around the tract exceeding the sham tract, and weight loss above 15%.

Prediction 1. If, with target engagement confirmed, intraosseous PRF produces no difference from sham insertion in any of hyperalgesia, trabecular architecture, osteoblastic surface fraction, and nerve measures, with confidence intervals excluding a standardized difference of 1.2 (the effect used for the sample size), the sensory-nerve mechanism does not operate in this OA model and the hypothesis fails this test. If structural effects persist after local sensory denervation, the mechanism is not nerve-mediated. If local NGF increases while pain behavior worsens, or if cartilage or synovitis scores deteriorate relative to sham, the therapeutic hypothesis fails for mechanically driven OA and the program does not advance.

### 6.3 Phase 0B: large-animal dosimetry and safety

Mice cannot reproduce human electrode-to-bone-volume ratios. A study in eight sheep or goats (four per timepoint) with naturally occurring or surgically induced OA will use a human-scale electrode to measure temperature at fixed distances from the tip, impedance, and field distribution, and to assess cortical entry-site integrity, osteonecrosis, and marrow injury by CT, MRI, and histology at 2 and 12 weeks. Phase 0B is a descriptive dosimetry and safety study, not powered for hypothesis testing; its size reflects the number of electrode tracts needed to characterize temperature profiles and tissue injury at two timepoints. The same stopping criteria apply.

### 6.4 Phase I: human feasibility and dosimetry

Twelve adults will be enrolled who have symptomatic medial knee OA, a WOMAC pain subscale score above 40 of 100, a medial tibial plateau BML of MOAKS size score 2 or 3 (or BLOKS grade 2 or 3), and no response to at least 3 months of conservative management. Electrodes will be placed under fluoroscopic or CT guidance using the dose specification from Stage 0A and Phase 0B. Outcomes: serious adverse events and procedure-related adverse events at 3 months, procedural success, *in vivo* impedance spectroscopy, and MRI at 3 months to assess the electrode tract, entry site, and subchondral integrity. Ethics approval and prospective registration in a primary clinical trial registry are prerequisites. Stopping rule: enrollment is suspended for independent safety review after any device-related or procedure-related serious adverse event. Event rates will be reported with exact 95% confidence intervals; with 12 participants and no events, the upper limit of the interval would be 26.5%, so Phase I can detect common harms but cannot establish safety.

Prediction 2. If any device-related serious adverse event occurs and the independent safety review attributes it to the PRF field rather than to needle access, or if impedance and dosimetry indicate that the field within 2 mm of the tip in living trabecular bone falls below the neuronal threshold established in Stage 0A (or, if that threshold is not available, below the provisional 330 V/cm benchmark), the physical rationale is not confirmed and the program does not advance without revising the dosimetry.

### 6.5 Phase II: external pilot randomized trial

A randomized external pilot trial with three arms: intraosseous PRF; a sham procedure with identical introducer and electrode placement through the cortex, the same duration and local anesthesia, and the generator connected but no energy delivered, to control for decompression and microfracture; and delayed treatment, unblinded and offered PRF after the 12-month visit. Participants in the PRF and sham arms and all outcome assessors are blinded. Allocation is 1:1:1 by central computer-generated randomization with concealed allocation, blinding success is assessed with a blinding index, and an independent data monitoring committee applies the Phase I stopping rule. Thirty-five participants per arm will be enrolled. This number follows recommendations that an external pilot measure at least 35 participants per group to estimate the standard deviation of a continuous outcome with adequate precision for planning a definitive trial (Level IV evidence, simulation study) (71). Phase II is not powered for efficacy; its purpose is to estimate the variance, the between-group difference with its confidence interval, recruitment, retention, and blinding success, which will determine the sample size of Phase III. The trial will be reported according to the CONSORT extension for randomized pilot and feasibility trials (72).

Primary structural outcome. BML volume at 6 months measured by semi-automated quantitative segmentation on fluid-sensitive sequences, read centrally by a radiologist blinded to allocation, with intra-reader and inter-reader reliability and the smallest detectable change, derived from test-retest limits of agreement, reported (7, 73). MOAKS BML size scores are reported as a secondary semiquantitative outcome (6). WORMS is not used to express percentage volume change, because WORMS grades each subregion on an ordinal 0–3 scale and an ordinal score cannot express a percentage change in volume.

Clinical outcomes. WOMAC pain, function, and total scores, patient global assessment (0–100), and KOOS pain subscale at 1, 3, 6, and 12 months, with OMERACT-OARSI responder status (74) derived at each visit, and MRI at 12 months. Follow-up to 12 months is required because intravenous zoledronic acid reduced BML area at 6 months, with only a trend at 12 months and no effect on BML size over 24 months in a larger trial (Level I–II evidence) (2, 75, 76); any PRF effect must be shown to persist.

Prediction 3. The structural hypothesis is supported if the between-group difference in BML volume change at 6 months favors PRF over sham and is maintained at 12 months. Because no minimal important change in BML volume has been established, Phase II reports the difference with its 95% confidence interval relative to the smallest detectable change of the measurement method. The smallest detectable change, derived from repeat MRI within 2 weeks in a subset of 15 participants, is an individual-level measure and is used here as a conservative threshold of structural relevance, not as a measure of group-level precision. The structural hypothesis is not supported if the confidence interval excludes a difference larger than the smallest detectable change.

Prediction 4. If the proportion of OMERACT-OARSI responders (74) at 3 months is not greater with PRF than with sham, with the 95% confidence interval of the difference excluding an absolute advantage of 15 percentage points, the prespecified progression criterion that would also serve as the target effect for powering Phase III, the analgesic effect is no greater than the effect of puncture, and the analgesic hypothesis fails in the knee.

How the measurements test the hypothesis. The hypothesis has three components: that PRF modulates the subchondral sensory nerve, that this modulation reduces pain, and that it shifts bone remodeling toward BML regression. Phase 0 tests the first component directly (target engagement, DRG activity, denervation arm) and links it to the second and third (pain behavior, bone and nerve histology). Phase II tests the second and third components in patients. Their joint pattern is interpreted as follows. Improvement in pain and in BML volume, preceded in Phase 0 by nerve-dependent changes abolished by denervation, supports the full hypothesis. Improvement in pain without structural change supports a neuromodulatory analgesic effect, similar in kind to genicular treatment, and requires withdrawal of the structural claim. Structural change without pain benefit argues against the sensory-nerve circuit as the dominant pain mechanism in this population. Beneficial effects in Phase 0 that persist after denervation indicate a mechanism other than the one proposed. These patterns are prespecified so that results cannot be fitted to the hypothesis after the fact.

### 6.6 Phase III: confirmatory trial

Conditional on Phase II supporting at least one component: a randomized, double-blind, sham-controlled, multicenter trial with sample size calculated from the Phase II variance and effect estimate, coprimary outcomes of BML volume change and WOMAC pain, and follow-up of at least 12 months.

## 7 Search methodology

A structured literature search was conducted in PubMed/MEDLINE on August 10, 2026, using (“pulsed radiofrequency” OR “pulsed radio frequency” OR “pulsed RF”) AND (intraosseous OR intra-osseous OR intramedullary OR subchondral OR “bone marrow” OR osteoblast OR osteoclast). The search was repeated on October 2, 2026 and retrieved six records; none concerned a PRF electrode within articular bone, and the only intraosseous PRF report was the osteonecrosis series (10). No study of a PRF electrode positioned within articular bone for OA or a BML was identified. A supplementary search for [“pulsed radiofrequency” AND (“bone marrow edema” OR “bone marrow lesion”)] retrieved one record, which concerned PEMF rather than PRF, and a search for (“pulsed radiofrequency” AND osteonecrosis) retrieved six records, of which only the cited series used an intraosseous PRF electrode. Supplementary searches covered intraosseous comparators (PRP, BMAC, subchondroplasty, basivertebral ablation), BML imaging and pharmacological treatment, PEMF and PRF dosimetry, and sensory nerve biology in bone (NGF, TrkA, ASIC3, EP4, skeletal interoception); the searches were updated during revision on October 2, 2026. This was a structured search rather than a systematic review and documents the basis of the novelty claim rather than providing a synthesis.

## 8 Limitations

This article presents a hypothesis, not clinical evidence. Most mechanistic elements derive from animal models or *in vitro* studies, and the human data are associations observed in resected osteoarthritic or osteonecrotic tissue; the only clinical experience with an intraosseous PRF electrode is one uncontrolled series of ten patients with osteonecrosis. Extrapolation from osteonecrosis to OA, from the hip to the knee, and from a single procedure to a therapeutic protocol has not been validated. The NGF paradox (Section 4) is an empirical question that requires Phase 0 data before human application.

The electric field distribution in living human trabecular bone at radiofrequency has not been measured. The 330 and 480 V/cm benchmarks derive from HEK-293 cells and are not validated biological thresholds for sensory neurons or bone. Marrow and mineralized bone have dielectric properties that differ from soft tissue, so bone-specific modeling and *ex vivo* dosimetry (Stage 0A) are prerequisites, not refinements.

The procedure requires image guidance, local anesthesia, and intraosseous access through an introducer needle. Risks include infection, cortical fracture at the entry site, marrow decompression or microfracture caused by insertion, thermal injury if temperature control fails, and vascular injury. These are comparable in category to the risks of intraosseous injection and subchondroplasty, but their magnitude for an intraosseous PRF electrode in the knee is unknown and must be monitored with explicit stopping rules (Section 6).

Small-animal models reproduce human BMLs incompletely, and the DMM model may not capture the mechanical drivers of human medial BMLs. A positive result in mice would justify, not replace, large-animal and human studies.

This manuscript proposes a research program and is not a clinical recommendation. The described procedure should not be performed outside an approved research protocol.

## 9 Conclusion

In murine models, sensory nerves in bone behave as bidirectional regulators of bone remodeling rather than as passive nociceptors. The osteoclast-nerve-osteoblast circuit it anchors is proposed here as a target for interventions directed at BMLs in knee OA, pending direct evidence that the circuit operates in osteoarthritic bone. In principle, an intraosseous PRF electrode can deliver a focal field to this circuit at intensities that external PEMF does not reach, and one small uncontrolled series indicates that intraosseous electrode placement in weight-bearing bone is technically feasible.

Whether this approach reduces BML volume and knee pain cannot be settled by argument or analogy. It requires bone-specific dosimetry, a Phase 0 study with functional neuronal readouts, targeted denervation, and safety endpoints that test the NGF paradox, large-animal dosimetry, and a sham-controlled pilot trial using quantitative BML volumetry. The predictions in Section 6 define success, failure, and inconclusive results at each stage. The program is worth pursuing only if the Phase 0 data support a sensory nerve mechanism under osteoarthritic conditions.

## Data Availability

The original contributions presented in the study are included in the article/Supplementary Material, further inquiries can be directed to the corresponding author.
